# A Doctor’s “Don’ts”

**Published:** 1890-04

**Authors:** 


### A DOCTOR'S "DON'TS."

Don't read in omnibusses or other jolting vehicles. Don't pick the teeth with pins or any other hard substance. Don't neglect any opportunity to ensure a variety of food. Don't eat and drink hot and cold things immediately in succession. Don't pamper the appetite with such variety of food as may lead to excess. Don't read, write, or do any delicate work unless receiving the light from the left side. Don't keep the parlor dark unless you value your carpet more than your children's health and your own. Don't endeavor to rest the mind by absolute inactivity ; let it seek its rest in other channels, and thus rest the tired part of the brain.

